# The Laminin-*α*1 Chain-Derived Peptide, AG73, Binds to Syndecans on MDA-231 Breast Cancer Cells and Alters Filopodium Formation

**DOI:** 10.1155/2019/9192516

**Published:** 2019-04-30

**Authors:** Madhavi Puchalapalli, Liang Mu, Chevaunne Edwards, Benjamin Kaplan-Singer, Pearl Eni, Kiran Belani, David Finkelstein, Arpan Patel, Megan Sayyad, Jennifer E. Koblinski

**Affiliations:** ^1^Department of Pathology, Virginia Commonwealth University, School of Medicine, Massey Cancer Institute, Richmond, VA, USA; ^2^Department of Pathology, Women's Cancer Research Program, Feinberg School of Medicine, Robert H. Lurie Comprehensive Cancer Institute, Northwestern University, Chicago, IL, USA; ^3^Craniofacial Developmental Biology and Regeneration Branch, National Institute of Dental and Craniofacial Research, National Institutes of Health, Department of Health and Human Services, Bethesda, MD, USA

## Abstract

Breast cancer is one of the most common forms of cancer affecting women in the United States, second only to skin cancers. Although treatments have been developed to combat primary breast cancer, metastasis remains a leading cause of death. An early step of metastasis is cancer cell invasion through the basement membrane. However, this process is not yet well understood. AG73, a synthetic laminin-*α*1 chain peptide, plays an important role in cell adhesion and has previously been linked to migration, invasion, and metastasis. Thus, we aimed to identify the binding partner of AG73 on breast cancer cells that could mediate cancer progression. We performed adhesion assays using MCF10A, T47D, SUM1315, and MDA-231 breast cell lines and found that AG73 binds to syndecans (Sdcs) 1, 2, and 4. This interaction was inhibited when we silenced Sdcs 1 and/or 4 in MDA-231 cells, indicating the importance of these receptors in this relationship. Through actin staining, we found that silencing of Sdc 1, 2, and 4 expression in MDA-231 cells exhibits a decrease in the length and number of filopodia bound to AG73. Expression of mouse Sdcs 1, 2, and 4 in MDA-231 cells provides rescue in filopodia, and overexpression of Sdcs 1 and 2 leads to increased filopodium length and number. Our findings demonstrate an intrinsic interaction between AG73 in the tumor environment and the Sdcs on breast cancer cells in supporting tumor cell adhesion and invasion through filopodia, an important step in cancer metastasis.

## 1. Introduction

Laminin-111 (LM-111) is involved in both normal and neoplastic breast biology and enhances adhesion, migration, and metastasis of tumor cells. This basement membrane glycoprotein is cleaved in the breast tumor environment, suggesting bioactive fragments may be released into the tumor environment [1–7]. Supporting this idea, synthetic peptides of LM-111 can alter the behavior of tumor cells. AG73 is a synthetic peptide based on the LM-*α*1 chain (RKRLQVQLSIRT, residues 2719-2731) that affects many physiological events. The role of AG73 in metastasis has been documented in melanoma, adenoid cystic, oral squamous cell, and ovarian carcinoma [8–14], and we previously reported that AG73 increases breast cancer metastasis to the bone [14]. Further, HT1080 human fibrosarcoma, B16F10 mouse melanoma, and SW480 human colon adenocarcinoma cells are known to adhere to AG73 [15]. However, a scrambled sequence of AG73 (LQQRRSVLRTKI) does not promote cell adhesion. Furthermore, AG73 can be conjugated to polysaccharides (chitosan and alginate) and hyaluronate hydrogels and promote cell viability, neurite extension, capillary-like networks with endothelial cells, and acinar-like structures with salivary gland cells [16–19]. AG73 also inhibits the ability of cells to spread on LM-111 [15], indicating it likely has physiological relevance.

The receptors for AG73 may also play an important role in tumor growth and metastasis. In the absence of an added peptide, a subpopulation of B16F10 cells, which were adhesion selected to AG73 over 30 times, has effects on tumor cells including increased invasion in vitro, subcutaneous tumor growth, and metastatic colony growth in the lungs and the liver [10]. These results suggest that AG73 receptors and their signaling pathway(s) are important in the metastasis of these cells, and thus, we focused on the receptors in the breast cancer cells that bind to AG73. AG73 specifically binds to cell surface proteoglycans, including syndecans (Sdcs) 1, 2, and 4 in nonbreast cells [12, 20–24]. The Sdcs are a family consisting of four transmembrane heparan sulfate proteoglycans that interact with integrins, growth factors, and chemokine receptors. Although they are not the primary receptors for the extracellular matrix (ECM), growth factors, or chemokines, they synergize with these molecules' prototypic receptors through simultaneous ligand engagement [25–27]. These receptors play critical regulatory roles in a variety of physiological and pathophysiological functions including wound healing, inflammation, neural patterning, tumor growth, and angiogenesis [28, 29]. Thus, we hypothesized that AG73 binds to breast cancer cells through the Sdcs. Here, we demonstrate that AG73-induced filopodium formation in breast cancer cells is mediated through Sdcs 1, 2, and 4. These data identify the receptors for a sequence in LM-111 that may play a role in the malignant phenotype of breast cancer. This is the first study to our knowledge to identify Sdcs as a binding partner for the laminin peptide, AG73, in breast cancer cells.

## 2. Materials and Methods

### 2.1. Cell Culture

The human breast carcinoma cell line MDA-MB-231 (MDA-231) was grown in DMEM/F-12 (Invitrogen) supplemented with 5% FBS (Gemini), 2 mM L-glutamine, 1 mM sodium pyruvate, 0.02 mM nonessential amino acids, puromycin (1 *μ*g/ml), and fungizone (2.5 *μ*l/ml) (Invitrogen). The MDA-231 cells expressing Green Fluorescent Protein (GFP) were a gift from Dr. D. Welch (The University of Kansas Medical Center). Human MCF7 and T47D breast carcinoma cells were grown in DMEM (Invitrogen) supplemented with 10% FBS (Gemini), 4.5 g/l D-glucose, (+) L-glut, and (-) sodium pyruvate (Invitrogen). Human SUM1315 breast carcinoma cells were grown in Ham's F-12 (Invitrogen), 5% FBS (Gemini), 5 *μ*g/ml insulin, 10 ng/ml EGF, and 10 mM HEPES (Sigma-Aldrich). Human MCF10A normal breast epithelial cells were grown in DMEM/F-12 (Invitrogen) supplemented with 5% horse serum (Invitrogen), 20 ng/ml EGF, 10 *μ*g/ml insulin, and 0.5 *μ*g/ml hydrocortisone (Sigma-Aldrich). All cell media contained 100 U/ml penicillin and 100 *μ*g/ml streptomycin (Invitrogen). Additionally, all cells were maintained at 37°C in a 5% CO_2_/95% humidified air atmosphere and were routinely checked for mycoplasma.

### 2.2. Adhesion Assays

Adhesion assays were performed as described [30, 31]. Briefly, 96-well plates were coated with AG73 and scrambled (2 *μ*g/ml), laminin (10 *μ*g/ml), rec-LG-4 and mutant rec-LG4 [20] (AG73 site mutated RKR-AAA; 11 *μ*g/ml, a kind gift from Drs. Kentaro Hozumi and Yoshihiko Yamada), or basement membrane extract (10 *μ*g/ml, BME/Matrigel, Trevigen/Bio-Techne) overnight in PBS at 4°C. (The scrambled peptide is a random sequence scramble of the AG73 amino acid sequence.) Wells were blocked with 3% heat-denatured BSA (10 min at 85°C) for 1 hr at 37°C. Cells were untreated or treated for 30 min with either PBS; 5 mM EDTA; 10 mg/ml heparin; heparan sulfate; chondroitin sulfates A, B, and C; hyaluronic acid (Sigma-Aldrich); 10-fold molar excess of AG73; or scrambled peptide, prior to 30 min adhesion. Heparin, heparan sulfate, and chondroitin sulfate B were used at 1-200 *μ*g/ml in the dose-response experiment with the IC_50_ calculated using Prism (GraphPad Software). The cells (30,000 cells/50 *μ*l/well of DMEM-0.1% BSA) were then added with the treatment to the blocked wells and incubated at 37°C for 30 min. The medium with unattached cells was removed from the wells. The adherent cells were stained for 10 min with 0.2% crystal violet in 20% methanol and washed twice with water. The cells were lysed with 10% SDS (50 *μ*l/well), and the optical density (600 nm) was measured. Each sample was performed in triplicate, and the experiment was repeated at least three times.

### 2.3. Silencing of Sdc Expression and Rescue

MDA-231 cells were infected with a lentivirus from Open Biosystems containing GFP. NS1 control is the nonsilencing (NS) shRNAmir sequence in the lentivirus vector GINZEO; Sdc 1 KD, the Sdc 1-silencing shRNAmir sequence in the lentivirus vector GINZEO; NS2 control, the nonsilencing (NS) shRNAmir sequence in the lentivirus vector GIPZ; Sdc 2 KD, the Sdc 2-silencing shRNAmir sequence in the lentivirus vector GIPZ; and Sdc 4 KD, the Sdc 4-silencing shRNAmir sequence in the lentivirus vector GIPZ. All cells were selected two times by flow cytometry for the top 5% of the GFP expression. These cells are not a cloned population but a heterogeneous population of cells that stably express the shRNAmir. Stable expression was determined by flow cytometry. To assure the results were not due to off-target effects of the shRNAmir, rescue experiments were carried out in the human cell lines using mouse Sdcs 1, 2, and 4 (OriGene) which are not targeted by the human shRNAmir. Specific targeting to human Sdc family members while expressing a mouse homolog has been demonstrated [32]. Mouse Sdc does rescue the knockdown of human Sdc. The mouse Sdc cDNA was cloned into the pBABE retrovirus (a kind gift from Dr. B. Parker, Northwestern University) which expresses the mCherry fluorescent protein. The cells were selected two times by flow cytometry for the top 5% of the mCherry fluorescent expression. Controls were the KD or NS cells infected with the pBABE retrovirus containing only the mCherry fluorescent protein and termed empty vector (EV) since there was no cDNA for Sdc. Stable expression was determined by flow cytometry.

### 2.4. Flow Cytometry

Cells were harvested with Versene (Invitrogen, 0.48 mM EDTA) for 10 min at 37°C with gentle agitation. Cells were washed in PBS and resuspended in cold buffer containing 1% FCS. A total of 2 × 10^5^ cells per sample was used. Following centrifugation, cells were resuspended in Hanks' balanced salt solution/2% BSA and incubated for 30 min at 4°C with titrated antibodies (20 *μ*g/ml mouse anti-Sdc 1 (B-A38, AbD Serotec), rabbit anti-Sdc 2 (R&D Systems), rabbit anti-Sdc 3 (midi), and rabbit anti-Sdc 4 (Zymed)); cells were then washed 2x with PBS, incubated with secondary antibody for 30 min at 4°C (1 : 100 dilution of donkey anti-mouse or rabbit-Cy5 (Jackson ImmunoResearch)), washed again 2x with PBS, and resuspended in 100 *μ*l of PBS. The samples were analyzed using a Beckman Coulter FC500 flow cytometer. Human Sdc expression was analyzed comparing the relative amount of human Sdc-stained cells with the IgG controls, and then KD cells were compared to NS-infected cells. Isotype-matched antibodies were used as negative controls.

### 2.5. Real-Time Quantitative PCR Analyses

Total RNA was isolated from breast cancer cells using RNAqueous-4PCR (Invitrogen) according to the manufacturer's instructions. RNA samples were then quantified using a NanoDrop (NanoDrop Technologies), and equal RNA concentrations were used for reverse-transcriptase PCR. First-strand cDNA synthesis and amplification were performed using a qScript cDNA SuperMix (Quanta BioSciences). Real-time PCR was carried out using a 7900HT Fast Real-Time PCR System (Applied Biosystems). cDNA templates were combined with a SYBR Green PCR Master Mix (Applied Biosystems). Primer sequences were obtained from PrimerBank, which contains primers that can be used for qPCR under stringent and allele-invariant amplification conditions [33]. The information can be accessed at https://pga.mgh.harvard.edu/primerbank/. The specific Sdc primers used were as follows: Sdc 1 forward: 5′-CTC TGA CAA CTT CTC CGG CTC-3′, Sdc 1 reverse: 5′-TCT GGC AGG ACT ACA GCC TC-3′; Sdc 2 forward: 5′-GCT CTG CCC CTA AAC TTC TGC-3′, Sdc 2 reverse: 5′-CTC TGC TGT GGT TTT GCT CCT-3′; Sdc 3 forward: 5′-CCC AGC TCC CTA GCT CTC TC-3′, Sdc 3 reverse: 5′-GCT GTC TCA ATG CCC GAC T-3′; Sdc 4 forward: 5′-GCT CTT CGT AGG CGG AGT-3′, Sdc 4 reverse: 5′-CCT CAT CGT CTG GTA GGG CT-3′; and GAPDH forward: 5′-TGG TGA AGC AGG CGT CGG AGG-3′, GADPH reverse: 5′-CGT CAA AGG TGG AGG AGT GGG TGT C-3′. Samples were amplified by an initial denaturation at 95°C for 2 min, followed by 40 cycles at 95°C for 10 sec and at 62°C for 30 sec. A melting curve was generated at the end of every run to ensure product uniformity. Gene expression was normalized to GAPDH. All reactions were run in triplicate, and each experiment was repeated at least three times.

### 2.6. Solid Phase

Solid-phase assays were performed as described [34]. Peptides were coated on 96-well plates (dried onto the well overnight, 100 *μ*g/ml in H_2_O); the wells were blocked (3% heat-denatured BSA) for 1 hr at 37°C and then incubated with MDA-231 cell lysate (2 mg/ml) in 3% BSA overnight at 4°C. For heparin inhibition, 20 mg/ml of heparin was added to the cell lysate before binding to the plate. For trypsin inhibition, cells were treated with trypsin (Invitrogen) for 10 min before lysing the cells. Binding was detected using anti-Sdc antibodies (0.2 *μ*g/ml, mouse anti-Sdc 1 (B-A38, AbD Serotec); rabbit anti-Sdcs 2, 3 (midi), and 4 (Zymed/Thermo Fisher Scientific)) followed by goat anti-mouse or rabbit-HRP-conjugated secondary antibodies (1 : 5000 dilution in 3%BSA-PBS). The signal was detected at 450 nm after incubation with TMB solution followed by 1 M H_2_SO_4_. For protein analysis, MDA-231 cell membranes were biotinylated (62) and membrane factions were isolated for enrichment of proteoglycans as described (82). Six-well plates were coated with peptide, and the biotinylated MDA-231 cell membrane was incubated with either AG73 or scrambled peptide. The bound material was scraped from the dish and electrophoresed on a 4-12% Bis-Tris gel. Bound material was transferred to PVDF and incubated with streptavidin-horseradish peroxidase, and reactive proteins were detected using a SuperSignal® West Dura Extended Duration Substrate (Pierce). Chemiluminescence was detected using a Fujifilm LAS-1000 Luminescent Image Analyzer “intelligent dark box” (Fujifilm Medical Systems USA Inc., Stamford, CT) using exposure times at subsaturation levels.

### 2.7. Actin Staining

AG73 or a scrambled peptide was dried onto coverslips overnight (100 *μ*g/ml in H_2_O). The coverslips were washed and blocked with 3% heat-denatured BSA. MDA-231 cells were collected using EDTA and counted and attached for 2 hours in serum-free media to the blocked and coated coverslips. The cells were fixed for 20 min in 4% paraformaldehyde (Electron Microscopy Sciences, Hatfield, PA) in PBS. After washing, the cells were permeabilized with 0.1% Triton X-100 (Sigma-Aldrich) in PBS for 5 min and washed, and then actin filaments were detected by staining with a 1 : 40 dilution of Alexa Fluor 647 fluorescent-labeled phalloidin (Invitrogen) in 1% BSA. The cells were then washed, fixed, and mounted upside down on slides with a SlowFade Antifade Reagent (Invitrogen) and observed with a LSM 510 Zeiss confocal microscope (Carl Zeiss MicroImaging Inc., Thornwood, NY). The filopodia were quantified using ImageJ analysis software.

## 3. Results and Discussion

### 3.1. AG73 Binds to Sdcs on Breast Cancer Cells

**Figure 1:**
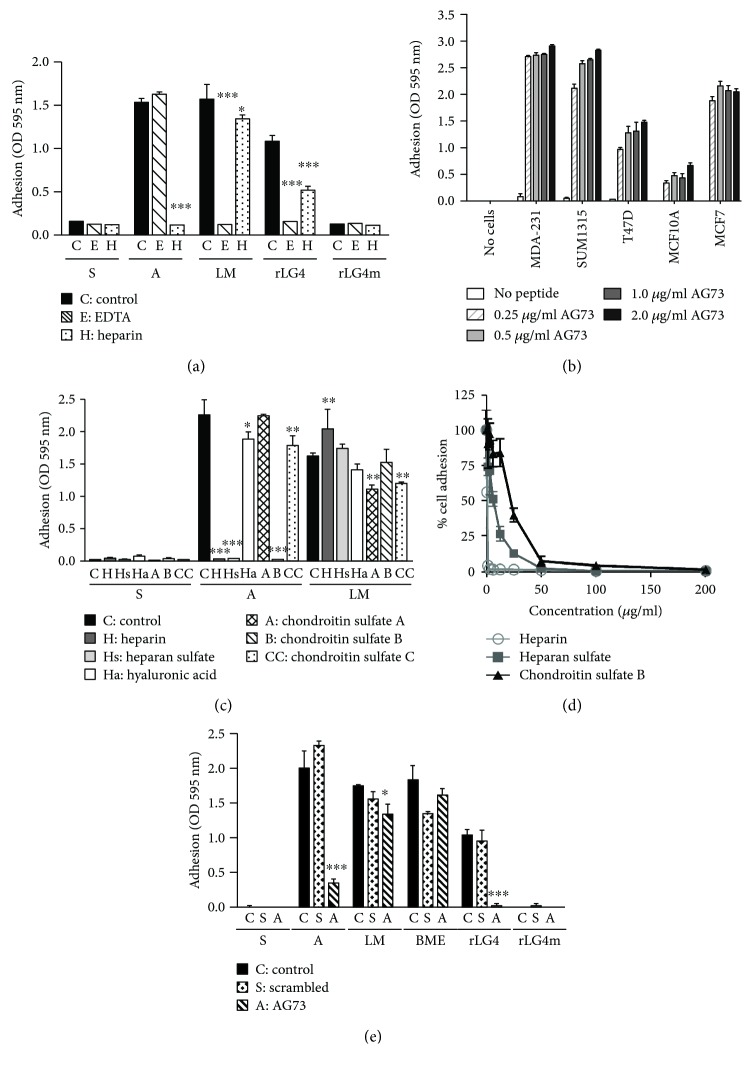
Breast cancer cell adhesion to AG73 is likely through a proteoglycan. (a) Adhesion to AG73 (A) and recombinant LG4 (rLG4) is inhibited by heparin (H, hatched; 10 mg/ml), while adhesion to LM-111 (LM) is inhibited by EDTA (E, diagonal; 5 mM) compared to PBS-treated cells (C, control, solid). The cells do not bind to the scrambled (S) peptide or rLG4 mutant (rLG4m, AG73 site mutated RKR-AAA). (b) Many different breast cancer cells (MDA-231, SUM1315, T47D, and MCF7) as well as normal breast cell lines (MCF10A) bind to AG73. (c) Heparin (H), heparan sulfate (Hs), and chondroitin sulfate B (B) significantly inhibited adhesion of MDA-231 cells to AG73 compared to PBS-treated cells (C, control). Hyaluronic acid (Ha), chondroitin sulfate A (A), and chondroitin sulfate C (CC) had no effect on adhesion to AG73 but did affect adhesion to LM-111 (LM). ^∗^ *p* < 0.05, ^∗∗^ *p* < 0.01, and ^∗∗∗^ *p* < 0.001. One-way ANOVA with Bonferroni posttest. (d) Serial dilutions of heparin, heparan sulfate, and chondroitin sulfate B inhibit adherence of cell binding. The IC_50_ followed the order heparin (0.8 *μ*g/ml), heparan sulfate (6.6 *μ*g/ml), and chondroitin sulfate B (22.4 *μ*g/ml);. (e) MDA-231 cell adhesion to LM-111 (LM), AG73, and rLG4 is inhibited by a 10-fold molar access of AG73 but not the scrambled (S) peptide. Adhesion to basement membrane extract (BME, Matrigel) was unaffected by AG73 or scrambled peptide. Cells were treated with either PBS (C, solid), 10-fold molar excess of AG73 (diagonal), or scrambled peptide (S, hatched) prior to adhesion. Bars: mean adhesion ± SEM.

AG73 mediates the adhesion of many different cell types, while a scrambled sequence of AG73 does not promote cell adhesion or have other biological activities [9, 30, 31]. To determine whether breast carcinoma cell lines bind to and interact with AG73, adhesion assays were performed as previously described [30, 31]. MDA-231 breast carcinoma cells bound to LM-111, AG73, and recombinant LM-LG4 (a recombinant protein from the laminin globular domain 4 at the C terminus of the LM-*α*1 chain where the AG73 sequence is located (rec-LG4) [20]), but not to either the scrambled peptide or the rec-LG4-mutant (mutated AG73 site; RKR-AAA [20]) (Figure 1(a)). We found that a variety of different breast cell lines bound to AG73 including MCF10A, T47D, SUM1315, and MDA-231 (Figure 1(b)).

To evaluate if cell binding was through proteoglycans or integrins, the effects of heparin and EDTA on MDA-231 cell adhesion to these peptides were examined. Heparin completely inhibited the binding of the MDA-231 cells to AG73, while EDTA had no effect (Figure 1(a)). These results suggest that the MDA-231 cells interact with AG73 through membrane-associated heparan sulfate proteoglycans but not through integrins. In addition, heparin and EDTA inhibited binding to LM-111 and rec-LG4 suggesting that cells bind to these molecules through proteoglycans and integrins (Figure 1(a)). Cell binding to LM-111 through both proteoglycans and integrin *α*6*β*1 is well established [35], and binding to the rec-LG4 is mediated through both Sdcs and integrin *α*2*β*1 in fibroblasts [20].

Additional experiments were performed to further characterize the proteoglycan receptor binding of AG73 to MDA-231 breast cancer cells. Since proteoglycans contain heparan sulfate and chondroitin sulfate side chains, these glycosaminoglycans (GAG) and others were tested for their ability to inhibit the MDA-231 cell attachment to AG73. Adhesion of MDA-231 cells to AG73 could be significantly (*p* < 0.001) blocked by heparin, heparan sulfate, and chondroitin sulfate B, but not by hyaluronic acid, chondroitin sulfate A, or chondroitin sulfate C (Figure 1(c)). Serial dilutions of heparin, heparan sulfate, and chondroitin sulfate B demonstrated that the concentration required to inhibit adherence by 50% followed the order of heparin (IC_50_, 0.8 *μ*g/ml), heparan sulfate (IC_50_, 6.6 *μ*g/ml), and chondroitin sulfate B (IC_50_, 22.4 *μ*g/ml) (Figure 1(d)). These results suggest that the charge of the glycosaminoglycan is important for preventing the AG73-receptor contact because heparin has the most negative charge and chondroitin sulfate B the least. In addition, these results indicate that heparan sulfate may be the GAG involved in binding AG73 since it had a greater affinity for AG73 than chondroitin sulfate B. Previous studies have also demonstrated that cell binding to AG73 can be inhibited by heparin and that these peptides bind to several different types of proteoglycans, e.g., AG73 binds to Sdcs 1 and 4 on both human salivary gland cells and human dermal fibroblasts and to a chondroitin-heparan sulfate proteoglycan on B16F10 melanoma cells [22, 30]. A tenfold molar access of AG73 was unable to inhibit breast cancer cell adhesion to the basement membrane extract (BME) that contains LM-111, entactin, collagen IV, and heparan sulfate proteoglycans [36] (Figure 1(e)). However, AG73 could inhibit the ability of the breast cancer cells to adhere to LM-111 (Figure 1(e)), indicating it likely has physiological relevance in the direct binding of cells to LM-111.

**Figure 2:**
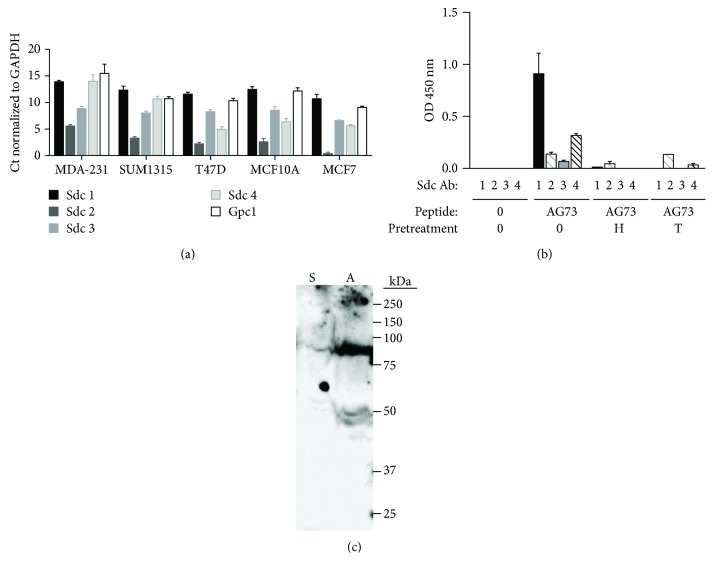
Sdcs 1-4 as well as glypican (Gpc)1 are expressed in MDA-231 cells. (a) qPCR was used to detect the message levels of each Sdc and glypican 1 (Gpc1) in breast cancer cell lines. Bars: mean Ct values are normalized to GAPDH ± SEM. All reactions were run in triplicate and repeated three times, and gene expression was normalized to the housekeeping gene GAPDH. (b) Sdcs 1 and 4 bind to AG73. For this solid-phase assay, wells were uncoated (0) or coated with AG73. Cells were pretreated with either heparin (H) or trypsin (T) before they were lysed. Controls were left untreated (0). The MDA-231 cell lysate binds to AG73 but not to the scrambled peptide (data not shown). The wells were then incubated with antibodies to Sdcs and detected using a secondary-HRP conjugate. Heparin and trypsin pretreatment inhibited Sdcs 1 and 4 binding to AG73. Bars: mean OD 450 nm ± SEM. (c) AG73 binds a >250, 90, and 50 kDa biotinylated protein from the surface of MDA-231 cells. Biotinylated MDA-231 cell membrane was incubated with AG73 (A) or scrambled peptide (S) as in (b). Proteoglycan bands usually appear as a smear due to the heparan sulfate and chondroitin sulfate glycosaminoglycan chains.

Hozumi et al. [20] have shown that the AG73 sequence is essential for binding of the proteoglycan receptors Sdcs 1, 2, and 4 to rec-LG4 on fibroblasts; however, other proteoglycans like glypican-1 do not bind to rec-LG4 or AG73. Thus, to explore if the Sdcs could be the proteoglycan receptor binding AG73 in breast cancer cells, expression of the Sdcs in MDA-231 cells was examined by qPCR. MDA-231 breast cancer cells as well as other breast cancer cell lines, e.g., MCF7, T47D, and SUM1315, express all four Sdcs, i.e., 1-4 (Figure 2(a)). Therefore, binding of the Sdcs to AG73 was investigated using a solid-phase assay as previously described [34]. Sdcs 1 and 4 bound to AG73 (Figure 2(b)) but did not bind to the scrambled peptide (data not shown), and this binding was inhibited by heparin and pretreatment of MDA-231 cells with trypsin, which cleaves proteoglycans from the cell surface [37, 38] (Figure 2(b)). CD44 or glypican-1 (other proteoglycan receptors) binding to either AG73 or the scrambled peptide was not detected (data not shown). To further characterize the receptor for AG73, the biotinylated MDA-231 cell membrane was incubated with either AG73 or scrambled peptide as in the solid-phase assay. Electrophoreses and blotting of the bound material revealed that AG73 binds components at >250, 90, and 50 kDa (Figure 2(c)). These molecular weights are consistent with those observed for the Sdc receptors [39, 40]. These data suggest that the likely candidates for the AG73 cell surface receptor on breast cancer cells are the Sdcs.

**Figure 3:**
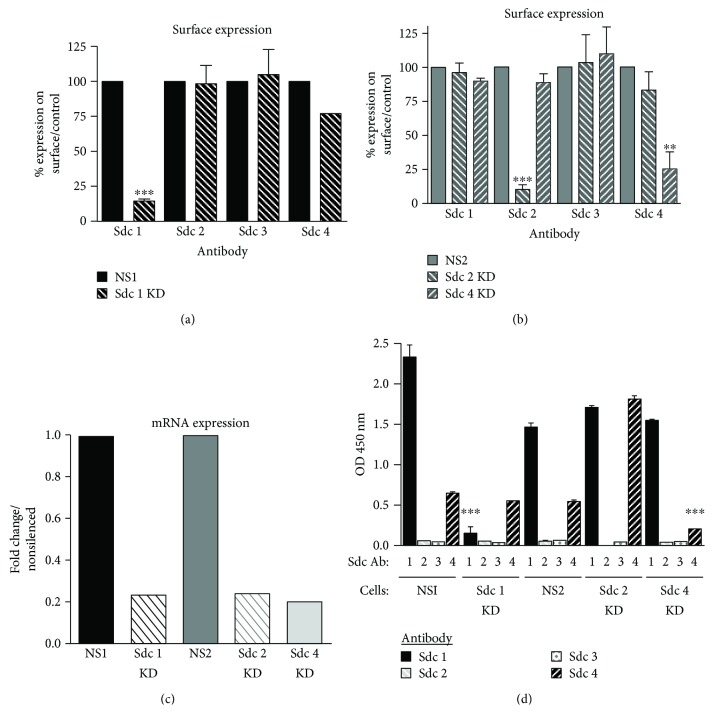
Expression of Sdcs 1, 2, and 4 is decreased by 75% at the protein (a, b) and message (c) levels in MDA-231 cells. (a, b) Flow cytometry measuring surface expression of Sdcs in MDA-231: (a) only Sdc 1 expression is decreased at the cell surface when the Sdc 1 expression is silenced in the MDA-231 cells. (b) In Sdc 2 KD cells, only the Sdc 2 surface expression is decreased, and in Sdc 4 KD cells, only the Sdc 4 surface expression is decreased. The decreased expression of these Sdcs is specific. (c) qPCR was used to detect the message levels of each Sdc. The fold change is plotted in comparison to the control. (d) Solid-phase assays were performed with the Sdc KD cells as described in Figure 2(b). Sdcs 1 and 4 bind to AG73 in control cells (NS1 and NS2) as well as the Sdc 2 KD cell lysate. No binding to AG73 was detected in the Sdc 1 and 4 KD cell lysate. Silencing of Sdcs 1 or 4 expression inhibited binding of these receptors to AG73, and thus, binding of the Sdc 1 and 4 antibodies was significantly decreased. Bars: mean OD 450 nm ± SEM. MDA-231-Sdc 1 KD cells compared to MDA-231-NS1 cells. MDA-231-Sdc 2 and 4 KD cells compared to MDA-231-NS2 cells. ^∗∗∗^ *p* < 0.001. One-way ANOVA with Bonferroni posttest.

Knockdown (KD) of Sdcs 1, 2, and 4 in the MDA-231 breast cancer cell line was generated using a shRNAmir. Sdc expression was successfully decreased by 75% both at the protein (Figures 3(a) and 3(b)) and message (Figure 3(c)) levels. Each knockdown was confirmed to be specific. The silencing of one family member did not affect the expression of the other family members (Figures 3(a) and 3(b)). In the solid-phase assay, knockdown of Sdc(s) 1 and/or 4 was found to inhibit binding to AG73 (Figure 3(d)), suggesting that these receptors are important in AG73-mediated cell activities. Sdcs 1, 2, and 4 have been shown to be a binding partner to AG73 in nonbreast cells including salivary gland cells, fibroblasts, and melanoma cells [12, 20–24]. Our results indicate that AG73 binds to Sdcs 1 and 4 in breast cancer cells. Sdcs 1 and 4 are expressed in the basolateral border of epithelial cells and myoepithelium in normal breast tissue [41]. In clinical samples of breast tumors, staining for Sdcs 1 and 4 is found in both the stroma and the tumor cells [41–43]. In addition, high Sdc 1 expression is associated with triple-negative and Her2+ breast tumors, which are highly metastatic subtypes, and it is a negative predictor of disease-free and overall survival [41–44]. These studies indicate that the Sdcs may play a role in breast cancer progression and metastasis. We have previously shown that AG73 increases breast cancer metastasis to the bone [14]. Taken together, our findings suggest that the interaction between AG73 and the Sdcs facilitates breast cancer metastasis.

### 3.2. AG73 Affects Filopodium Formation in Breast Cancer Cells through Sdcs 1, 2, and 4

**Figure 4:**
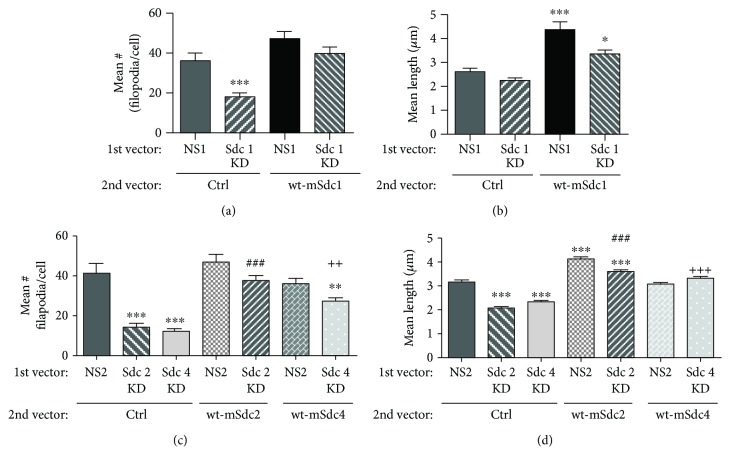
Silencing the expression of Sdcs 1, 2, and 4 decreased filopodium formation in MDA-231 breast cancer cells. (a) Silencing of the Sdc 1 expression decreased the number of filopodia/cells, and wild-type-mouse Sdc 1 (wt-mSdc1) could rescue this significant decrease. (b) The silencing of the Sdc 1 expression had no effect on the length of filopodia; however, overexpression of wt-mSdc1 (NS1-wt-mSdc1) as well as the rescue of the Sdc 1 knockdown increased the length of the filopodia. (c, d) Silencing of the Sdc 2 and 4 expression decreased the number of filopodia/cells (c) and the length of the filopodia (d). Expression of wt-mSdc2 or wt-mSdc4, respectively, could rescue these decreases. Overexpression of Sdc 2 (NS2-wt-mSdc2) but not Sdc 4 (NS2-wt-mSdc4) increased the length of the filopodia. Images in Supplemental Figure 1. ^∗∗∗^ *p* < 0.001 and ^∗∗^ *p* < 0.01 comparing NS2 to all other conditions; ^###^ *p* < 0.001 comparing Sdc 2 KD to Sdc 2 KD rescued with wt-mSdc2; ^++^ *p* < 0.01 or ^+++^ *p* < 0.001 comparing Sdc 4 KD to Sdc 4 KD rescued with wt-mSdc2. One-way ANOVA with Bonferroni posttest.

Filopodia play key roles in cancer cell migration, invasion, and metastasis [45]. We previously demonstrated that AG73 increases the formation of filament spikes in breast cancer cells, which resemble filopodia, whereas a scrambled peptide does not cause these morphological changes [14]. These increased filopodia are also seen in fibroblasts bound to AG73 [31]. Silencing of the expression of Sdcs 1, 2, or 4 significantly decreased the length and number of filopodia on MDA-231 breast cancer cells bound to AG73 (Figure 4 and Supplemental Figure 1). Expression of mouse Sdcs 1, 2, or 4, in the silenced cells, could rescue this decrease in filopodium length and number. Furthermore, overexpression of Sdcs 1 and 2 significantly increased the length of filopodia on the cells (Figure 4 and Supplemental Figure 1). These data demonstrate that AG73 binds to Sdcs 1, 2, and 4 on breast cancer cells and mediates filopodium formation through these Sdcs. Although we could not detect Sdc 2 in the solid-phase assay possibly due to limitations with antibody recognition in this assay, we did however still observe its effects on filopodium formation. A previous study also reported a synergistic relationship between AG73, Sdcs, and integrins in promoting cell adhesion and spreading, thus supporting our findings reported here [16]. The increase in filopodia we observed in our study emphasizes a possible link between AG73, Sdcs, and cancer as others have shown that expression of filopodium regulatory proteins in cancer patients correlates with poor prognosis and low survival [45]. In addition, a meta-analysis of filopodium gene expression in breast cancer patients revealed a link between filopodium-inducing genes and high rates of breast cancer metastasis [46]. Overall, our findings demonstrate a critical function resulting from the interaction between AG73 and the Sdcs in driving filopodium formation in breast cancer cells.

## 4. Conclusions

Breast cancer metastasis affects 20-30% of patients and remains to be fully understood [47]. In order to metastasize, disseminated breast cancer cells must cooperate with their environment to bypass the basement membrane and enter into circulation to transit to distant sites. In this study, we focused on this early step in cancer progression and thus investigated the interaction between breast cancer cells and AG73, a laminin peptide found in the basement membrane. We focused on receptors for AG73 and determined that the heparan sulfate proteoglycan receptors, Sdcs 1, 2, and 4, expressed on various breast cancer cells, facilitate this interaction by binding to AG73. We believe this to be the first report describing a direct binding relationship between AG73 and the Sdcs in breast cancer cells. Further, we found that the binding of AG73 to Sdcs 1, 2, and 4 promotes filopodium formation in breast cancer cells such as the MDA-231 cell line. Increased filopodia in breast cancer cells have been associated with tumor invasion and metastasis [45, 46]. Taken together, our findings demonstrate a previously unreported link between AG73 and Sdcs 1, 2, and 4 on breast cancer cells, highlighting the unique interplay between cancer cells and the tumor environment in mediating cancer progression.

## Data Availability

The data used to support the findings of this study are included within the article and supplementary figure.

## Supplementary Materials

Supplementary Materials Supplemental Figure 1: silencing the expression of Sdcs 1, 2, and 4 decreased filopodium formation in MDA-231 breast cancer cells and wtSdc (WT) can rescue filopodium formation. Representative images are included in the quantification shown in Figure 4. Cells infected with the nonsilencing (NS) shRNAmir as well as the empty vector (EV) were used as the control. This EV was the same vector (pBABE) that was used for the wtSdc expression.

## References

[1] Ioachim E., Charchanti A., Briasoulis E. (2002). Immunohistochemical expression of extracellular matrix components tenascin, fibronectin, collagen type IV and laminin in breast cancer: their prognostic value and role in tumour invasion and progression. *European Journal of Cancer*.

[2] Zheng W. Q., Looi L. M., Cheah P. L. (2002). Correlation between laminin and cathepsin D expressions in breast carcinoma. *Tumori Journal*.

[3] Ioachim E., Kamina S., Kontostolis M., Agnantis N. J. (1997). Immunohistochemical expression of cathepsin D in correlation with extracellular matrix component, steroid receptor status and proliferative indices in breast cancer. *Virchows Archiv*.

[4] Albrechtsen R., Nielsen M., Wewer U., Engvall E., Ruoslahti E. (1981). Basement membrane changes in breast cancer detected by immunohistochemical staining for laminin. *Cancer Research*.

[5] Bissell M. J., Rizki A., Mian I. S. (2003). Tissue architecture: the ultimate regulator of breast epithelial function. *Current Opinion in Cell Biology*.

[6] Gudjonsson T., Ronnov-Jessen L., Villadsen R., Rank F., Bissell M. J., Petersen O. W. (2002). Normal and tumor-derived myoepithelial cells differ in their ability to interact with luminal breast epithelial cells for polarity and basement membrane deposition. *Journal of Cell Science*.

[7] Kikkawa Y., Hozumi K., Katagiri F., Nomizu M., Kleinman H. K., Koblinski J. E. (2013). Laminin-111-derived peptides and cancer. *Cell Adhesion & Migration*.

[8] Nascimento C. F., de Siqueira A. S., Pinheiro J. J. V., Freitas V. M., Jaeger R. G. (2011). Laminin-111 derived peptides AG73 and C16 regulate invadopodia activity of a human adenoid cystic carcinoma cell line. *Experimental Cell Research*.

[9] Kim W. H., Nomizu M., Song S. Y. (1998). Laminin‐*α*1‐chain sequence Leu‐Gln‐Val‐Gln‐Leu‐Ser‐Ile‐Arg (LQVQLSIR) enhances murine melanoma cell metastases. *International Journal of Cancer*.

[10] Song S. Y., Nomizu M., Yamada Y., Kleinman H. K. (1997). Liver metastasis formation by laminin‐1 peptide (LQVQLSIR)‐adhesion selected B16‐F10 melanoma cells. *International Journal of Cancer*.

[11] Yoshida Y., Hosokawa K., Dantes A., Kotsuji F., Kleinman H. K., Amsterdam A. (2001). Role of laminin in ovarian cancer tumor growth and metastasis via regulation of Mdm2 and Bcl-2 expression. *International Journal of Oncology*.

[12] Suzuki N., Ichikawa N., Kasai S. (2003). Syndecan binding sites in the laminin *α*1 chain G domain. *Biochemistry*.

[13] Siqueira A. S., Gama-de-Souza L. N., Arnaud M. V. C., Pinheiro J. J. V., Jaeger R. G. (2010). Laminin-derived peptide AG73 regulates migration, invasion, and protease activity of human oral squamous cell carcinoma cells through syndecan-1 and *β*1 integrin. *Tumour Biology*.

[14] Engbring J. A., Hossain R., VanOsdol S. J. (2008). The laminin *α*-1 chain derived peptide, AG73, increases fibronectin levels in breast and melanoma cancer cells. *Clinical & Experimental Metastasis*.

[15] Nomizu M., Kim W. H., Yamamura K. (1995). Identification of cell binding sites in the laminin *α*1 chain carboxyl-terminal globular domain by systematic screening of synthetic peptides. *Journal of Biological Chemistry*.

[16] Hozumi K., Akizuki T., Yamada Y. (2010). Cell adhesive peptide screening of the mouse laminin *α*1 chain G domain. *Archives of Biochemistry and Biophysics*.

[17] Mochizuki M., Philp D., Hozumi K. (2007). Angiogenic activity of syndecan-binding laminin peptide AG73 (RKRLQVQLSIRT). *Archives of Biochemistry and Biophysics*.

[18] Yamada Y., Hozumi K., Aso A. (2012). Laminin active peptide/agarose matrices as multifunctional biomaterials for tissue engineering. *Biomaterials*.

[19] Yamada Y., Hozumi K., Katagiri F., Kikkawa Y., Nomizu M. (2013). Laminin-111-derived peptide-hyaluronate hydrogels as a synthetic basement membrane. *Biomaterials*.

[20] Hozumi K., Suzuki N., Nielsen P. K., Nomizu M., Yamada Y. (2006). Laminin *α*1 chain LG4 module promotes cell attachment through syndecans and cell spreading through integrin *α*2*β*1. *Journal of Biological Chemistry*.

[21] Hoffman M. P., Engbring J. A., Nielsen P. K. (2001). Cell type-specific differences in glycosaminoglycans modulate the biological activity of a heparin-binding peptide (RKRLQVQLSIRT) from the G domain of the laminin *α*1 chain. *Journal of Biological Chemistry*.

[22] Engbring J. A., Hoffman M. P., Karmand A. J., Kleinman H. K. (2002). The B16F10 cell receptor for a metastasis-promoting site on laminin-1 is a heparan sulfate/chondroitin sulfate-containing proteoglycan. *Cancer Research*.

[23] Gama-de-Souza L. N., Cyreno-Oliveira E., Freitas V. M. (2008). Adhesion and protease activity in cell lines from human salivary gland tumors are regulated by the laminin-derived peptide AG73, syndecan-1 and *β*1 integrin. *Matrix Biology*.

[24] Hozumi K., Kobayashi K., Katagiri F., Kikkawa Y., Kadoya Y., Nomizu M. (2010). Syndecan- and integrin-binding peptides synergistically accelerate cell adhesion. *FEBS Letters*.

[25] Bass M. D., Morgan M. R., Humphries M. J. (2009). Syndecans shed their reputation as inert molecules. *Science Signaling*.

[26] Lopes C. C., Toma L., Pinhal M. A. S. (2006). EJ-*ras* oncogene transfection of endothelial cells upregulates the expression of syndecan-4 and downregulates heparan sulfate sulfotransferases and epimerase. *Biochimie*.

[27] Tkachenko E., Rhodes J. M., Simons M. (2005). Syndecans: new kids on the signaling block. *Circulation Research*.

[28] Alexopoulou A. N., Multhaupt H. A. B., Couchman J. R. (2007). Syndecans in wound healing, inflammation and vascular biology. *The International Journal of Biochemistry & Cell Biology*.

[29] Morgan M. R., Humphries M. J., Bass M. D. (2007). Synergistic control of cell adhesion by integrins and syndecans. *Nature Reviews Molecular Cell Biology*.

[30] Hoffman M. P., Nomizu M., Roque E. (1998). Laminin-1 and laminin-2 G-domain synthetic peptides bind syndecan-1 and are involved in acinar formation of a human submandibular gland cell line. *Journal of Biological Chemistry*.

[31] Suzuki N., Nakatsuka H., Mochizuki M. (2003). Biological activities of homologous loop regions in the laminin *α* chain G domains. *Journal of Biological Chemistry*.

[32] Beauvais D. L. M., Rapraeger A. C. (2004). Syndecans in tumor cell adhesion and signaling. *Reproductive Biology and Endocrinology*.

[33] Wang X., Spandidos A., Wang H., Seed B. (2011). PrimerBank: a PCR primer database for quantitative gene expression analysis, 2012 update. *Nucleic Acids Research*.

[34] Ponce M. L., Nomizu M., Kleinman H. K. (2001). An angiogenic laminin site and its antagonist bind through the *α*v*β*3 and *α*5*β*1 integrins. *The FASEB Journal*.

[35] Ekblom P., Lonai P., Talts J. F. (2003). Expression and biological role of laminin-1. *Matrix Biology*.

[36] Benton G., Arnaoutova I., George J., Kleinman H. K., Koblinski J. (2014). Matrigel: from discovery and ECM mimicry to assays and models for cancer research. *Advanced Drug Delivery Reviews*.

[37] Bame K. J. (1993). Release of heparan sulfate glycosaminoglycans from proteoglycans in Chinese hamster ovary cells does not require proteolysis of the core protein. *Journal of Biological Chemistry*.

[38] Heinegård D., Hascall V. C. (1974). Aggregation of cartilage proteoglycans III. Characteristics of the proteins isolated from trypsin digests of aggregates. *Journal of Biological Chemistry*.

[39] Granés F., García R., Casaroli-Marano R. P. (1999). Syndecan-2 induces filopodia by active cdc42Hs. *Experimental Cell Research*.

[40] Mundhenke C., Meyer K., Drew S., Friedl A. (2002). Heparan sulfate proteoglycans as regulators of fibroblast growth factor-2 receptor binding in breast carcinomas. *The American Journal of Pathology*.

[41] Baba F., Swartz K., van Buren R. (2006). Syndecan-1 and syndecan-4 are overexpressed in an estrogen receptor-negative, highly proliferative breast carcinoma subtype. *Breast Cancer Research and Treatment*.

[42] Barbareschi M., Maisonneuve P., Aldovini D. (2003). High syndecan-1 expression in breast carcinoma is related to an aggressive phenotype and to poorer prognosis. *Cancer*.

[43] Leivonen M., Lundin J., Nordling S., von Boguslawski K., Haglund C. (2004). Prognostic value of syndecan-1 expression in breast cancer. *Oncology*.

[44] Nguyen T. L., Grizzle W. E., Zhang K., Hameed O., Siegal G. P., Wei S. (2013). Syndecan-1 overexpression is associated with nonluminal subtypes and poor prognosis in advanced breast cancer. *American Journal of Clinical Pathology*.

[45] Jacquemet G., Paatero I., Carisey A. F. (2017). FiloQuant reveals increased filopodia density during breast cancer progression. *The Journal of Cell Biology*.

[46] Arjonen A., Kaukonen R., Ivaska J. (2014). Filopodia and adhesion in cancer cell motility. *Cell Adhesion & Migration*.

[47] American Cancer Society (2015). *Breast Cancer Facts & Figures 2015-2016*.

