# Supplementary figures and images for: The Laminin-α1 Chain-Derived Peptide, AG73, Binds to Syndecans on MDA-231 Breast Cancer Cells and Alters Filopodium Formation
Source: Anal Cell Pathol (Amst). 2019 Apr 30;2019:9192516. doi: 10.1155/2019/9192516 (PMC6515157; doi:10.1155/2019/9192516)

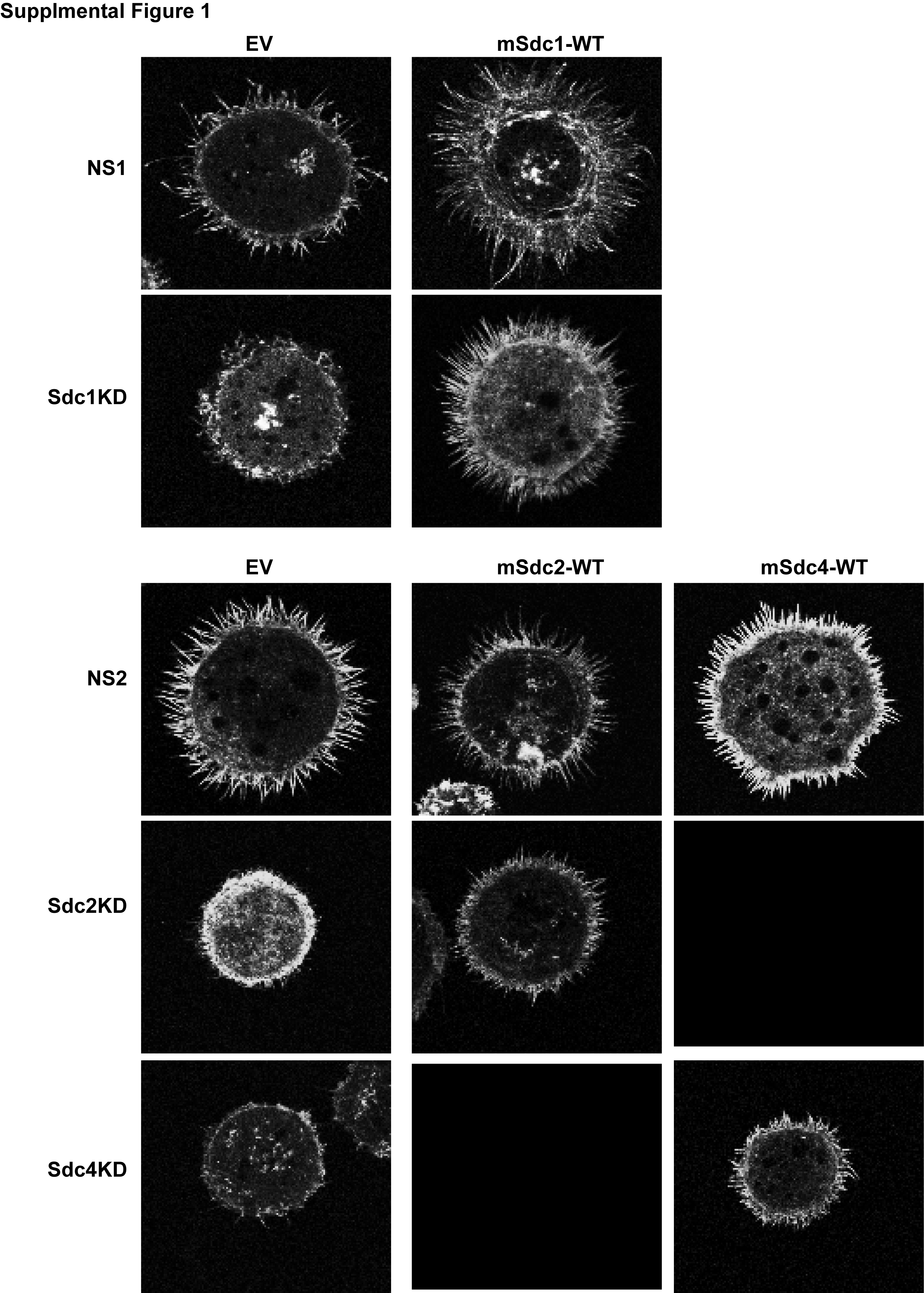

Supplement: Supplementary Materials — Supplemental Figure 1: silencing the expression of Sdcs 1, 2, and 4 decreased filopodium formation in MDA-231 breast cancer cells and wtSdc (WT) can rescue filopodium formation. Representative images are included in the quantification shown in Figure 4. Cells infected with the nonsilencing (NS) shRNAmir as well as the empty vector (EV) were used as the control. This EV was the same vector (pBABE) that was used for the wtSdc expression. [file 9192516.f1.tif]
